# *Prosopis juliflora* leave extracts induce cell death of MCF-7, HepG2, and LS-174T cancer cell lines

**DOI:** 10.17179/excli2020-2830

**Published:** 2020-09-09

**Authors:** Serag Eldin I. Elbehairi, Ahmed Ezzat Ahmed, Ali A. Alshati, Mohammed A. Al-Kahtani, Mohammad Y. Alfaifi, Khalid M. Alsyaad, Ali Yahya A. Alalmie, Mohammed M. Elimam Ahamed, Mahmoud F. Moustafa, Sadeq K. Alhag, Ahmed M. Al-Abd, Ahmed M. Abbas

**Affiliations:** 1 Department of Biology, College of Science, King Khalid University, Abha, Saudi Arabia; 2 Cell Culture Laboratory, Egyptian Organization for Biological Products and Vaccines (VACSERA Holding Company), Agouza, Giza, Egypt; 3 Department of Theriogenology, Faculty of Veterinary Medicine, South Valley University, Qena, Egypt; 4 Director of the Research Centre, College of Science, King Khalid University, Abha, Saudi Arabia; 5 The Poison Control and Medical Forensic Chemistry Centre, Asir, Saudi Arabia; 6 Department of Chemistry, College of Science, King Khalid University, Abha, Saudi Arabia; 7 Unit of Bee Research and Honey Production, College of Science, King Khalid University, Abha, Saudi Arabia; 8 Department of Botany & Microbiology, Faculty of Science, South Valley University, Qena, Egypt; 9 Biology Department, College of Arts and Science, King Khalid University, Muhayl Asir, Saudi Arabia; 10 Biology Department, College of Science, Ibb University, Yemen; 11 Department of Pharmaceutical Science, College of Pharmacy & Thumbay Research Institute of Precision Medicine, Gulf Medical University, Ajman, UAE; 12 Pharmacology Department, Medical Division, National Research Centre, Cairo, Egypt

**Keywords:** Prosopis juliflora, cytotoxicity, apoptosis, cancer, MCF-7, HePG2, LS-174T

## Abstract

*Prosopis juliflora* (*P. juliflora*) is a widespread phreatophytic tree, which belongs to the Fabaceae family. The goal of the present study is to investigate the potential anti-cancer effect of *P. juliflora *leave extracts and to identify its chemical composition. For this purpose, MCF-7 (breast), HepG2 (liver), and LS-174T (colorectal) cancer cell lines were cultivated and incubated with various concentrations of *P. juliflora *leave extracts, and its impact on cell viability, proliferation, and cell cycle stages was investigated. *P. juliflora *leave extracts induced concentration-dependent cytotoxicity against all tested cancer cell lines. The calculated IC_50_ was 18.17, 33.1 and 41.9 μg/ml for MCF-7, HePG2 and LS-174T, respectively. Detailed analysis revealed that the cytotoxic action of *P. juliflora *extracts was mainly via necrosis but not apoptosis. Moreover, DNA content flow cytometry analysis showed cell-specific anti-proliferative action and cell cycle stages arrest. In order to identify the anti-cancer constituents of *P. juliflora*, the ethyl extracts were analyzed by liquid chromatography-mass spectrometry. The major constituents identified in the ethyl extracts of *P. juliflora* leaves were hydroxymethyl-pyridine, nicotinamide, adenine, and poly-(methyl methacrylate) (PMMA). In conclusion, *P. juliflora* ethyl acetate extracts have a potential anti-cancer effect against breast adenocarcinoma, hepatocellular carcinoma, and colorectal adenocarcinoma, and is enriched with anti-cancer constituents. See also Figure 1(Fig. 1).

## Introduction

*Prosopis juliflora* is a widespread plant belonging to the Fabaceae family. It is also known as mesquite or velvet mesquite or Ghaf Bahri, and is commonly native to Southwest United States, Northwest Mexico, and invasive in many tropical and subtropical regions, including North Africa and the Arab Gulf region (Abbas et al., 2016[1]). The tree is approximately 8-12 meter (39 ft.) height, with a wide trunk up to 1.2 meter (3.9 ft.) diameter (Valdivia, 1972[36]). The World Health Organization (WHO) estimated that about 80 % of humans still depend on herbal medication for treatment of diseases due to several reasons including cost, availability and less side effects compared to synthetic drugs (Ukande et al., 2019[35]). The genus *Prosopis* (Fabaceae) includes 44 species and is considered among the most damaging invasive species in the world (Persia et al., 2016[24]). Most of the *Prosopis *species (*P. africana, P. alba, P. cineraria, P. farcta, P. glandulosa, P. juliflora, P. nigra, P. ruscifolia and P. spicigera)* are effectively used for treatment of several diseases (Persia et al., 2016[24]), including respiratory, digestive, urinary and obstetrical affections (Sharifi-Rad et al., 2019[31]). The *P. juliflora* contains phytochemicals rich in alkaloids, phenolic compounds (Prabha et al., 2014[25]), and flavonoids in particular (Diarra et al., 2015[5]; Catarino et al., 2016[3]). Several studies have reported the pharmaceutical activities of *P. juliflora* leaves, such as antibacterial (Catarino et al., 2016[3]; Dos Santos et al., 2013[7]), antifungal (Raghavendra et al., 2009[28]), antipyretic (Gopinath et al., 2013[11]), antiulcer (Jagan Mohan Reddy et al., 2014[16]) and antiprotozoal activities (Garbi et al., 2014[9]). Moreover, other studies reported the potency of *P. juliflora* extract in inhibiting cancer cell growth (Sathiya and Muthuchelian, 2011[29]). Recent studies have shown variant cytotoxic potencies of *P. juliflora* extracts against the breast/MCF-7 (Gurushidhappa et al., 2018[12]) or the liver/HePG2 (Hassan et al., 2019[15]) cancer cells showing strong cytotoxic effect. On the other hand, no study investigated the effects of *P. juliflora* on the colorectal cancer cell line, but other *Prosopis* species, i.e. *P. strombulifera* extract had evidenced cytotoxicity against the colorectal in a dose-dependent manner (Persia et al., 2016[24]; Cattaneo et al., 2014[4]). Thus, the present study aimed to; 1) investigate the potential anti-cancer effect of *P. juliflora* ethyl acetate extract on the human breast, liver and colorectal cancer cell lines; MCF-7, HepG2 and LS-174T, respectively, and compare the characteristics of cytotoxic potencies in those cells with a special reference to the cell cycle arrest, and 2) identify the chemical constituents of the plant leaves extract.

## Materials and Methods

### Preparation of ethyl acetate extract of Prosopis juliflora leaves 

The leaves of *P. juliflora* were freshly collected from mature plants grown in Aqbet Dalaa, Asir region, south-west Saudi Arabia (17°53.863˝N-42°24.429˝E) in January. The collected leaves were dried in an oven at 40 °C until a constant weight was obtained. The dried leaves were crushed into fine pieces, and then ground with a mill grinder into powder form. Ethyl acetate (Friedemann Schmidt, Parkwood, WA, USA) was used as organic solvent for extraction of the plant constituents, as following: a) in a sterile flask, 500 ml of 70 % aqueous ethanol was mixed with 250 g of the powdered leaves and then left in water bath at 40 °C for 1 h; b) the mixture was kept on stirrer for 7 days at 18-24 °C in the dark for thorough maceration; c) the suspension was filtered using Whatman paper (Thermo Fisher Scientific, Inc., Waltham, MA, USA), and then evaporated using a rotary evaporator (Ika, Deutschland, Germany) to obtain crude ethyl acetate extracts and to separate the solvent; d) the extract was left at room temperature (20-26 °C) for complete evaporation for 5 days. The crude extract weighed 3.723 g, and the extraction yield was 30 % of the ethyl acetate yield; and finally e) 10 mg of the crude extract was diluted with 100 µl of dimethyl sulfoxide (DMSO) as a stock solution for bioactivity assays.

### Cancer cell lines

Adenocarcinoma (MCF-7), hepatocellular carcinoma (HepG2), and colorectal cancer (LS-174T) cell lines were obtained from American Type Culture Collection (ATCC) (Manassas, VA, USA). Those cell lines were cultured in the specific RPMI-1640 media containing penicillin (100 U/ml); streptomycin (100 µg/ml), and heat-inactivated fetal bovine serum (FBS; 10 %). The cells were propagated in a humidified cell culture incubator with 5 % (v/v) CO_2_ at 37 °C. 

### Cytotoxicity assay

The ethyl acetate extract of *P. juliflora* was tested for the cytotoxicity of MCF-7, HepG2 and LS-174T by using Sulforhodamine-B (SRB) assay (Skehan et al., 1990[33]). The exponentially growing cells were collected using 0.25 % Trypsin-EDTA and seeded in 96-well plates as 2000 cells/well. Cells were treated with the plant extract for 72 h and subsequently fixed with trichloroacetic acid (TCA; 10 %) at 4 °C for 1 h. After several washings with distilled water, the cells were exposed to 0.4 % SRB for 10 min at room temperature in a dark place and subsequently washed with 1 % glacial acetic acid. After the plates dried overnight, Tris-HCl was used to dissolve the SRB stained cells. Color intensity was measured at 540 nm with a Spectra-Max® ELISA microplate reader (Molecular Devices LLC, San Jose, CA, USA). Finally, the half maximal inhibitory concentration (IC_50_) was calculated for the plant extract against each cell line.

### Cell cycle analysis 

Effect of the plant extract on the cell cycle distribution of MCF-7, HepG2 and LS-174T cells was determined by using the flow cytometry analysis (FCA). The cells were subjected to the pre-determined IC_50_ of the free media (control) or *P. juliflora* ethyl acetate extract-treated media for 48 h. After incubation, the cells were collected by trypsinization and washed twice with ice-cold PBS and re-suspended in 0.5 ml of PBS. Two milliliters of 60 % ice-cold ethanol were added gently while vortexing and cells were incubated for fixation at 4 °C for 1 h. Upon analysis, the fixed cells were washed and resuspended in 1 ml of PBS containing 50 μg/ml RNase A and 10 μg/ml propidium iodide (PI). After 20 min of incubation in darkness at 37 °C, the cells were analyzed for DNA content using FL2 (λex/em 535/617 nm) signal detector (ACEA Novocyte™ flow cytometer (ACEA Biosciences Inc., San Diego, CA, USA). About 12,000 events occurred per sample. Cell cycle distribution was calculated using ACEA Novo-Express™ software (ACEA Biosciences Inc., San Diego, CA, USA).

### Apoptosis assay

Effect of *P. juliflora* ethyl acetate extract on apoptosis and necrosis of the studied cell lines was determined by flow cytometry analysis using Annexin/V-FITC apoptosis detection kit, according to the manufacturer's instructions (Abcam Inc., Cambridge Science Park, Cambridge, UK). Briefly, the different cell lines were treated with the respective IC_50_ of the plant extract for 48 h. Subsequently; the cells were collected by trypsinization, washed twice with ice-cold PBS, and re-suspended in 0.5 ml of annexin/V-FITC/PI solution for 30 min in dark according to the manufacturer's protocol. After staining at room temperature, the cells were injected into the ACEA Novocyte™ FCA (ACEA Biosciences Inc., San Diego, CA, USA) and analyzed for FITC and propidium iodide (PI) fluorescent signals using FL1 and FL2 detectors, respectively (λex/em 488/530 nm for FITC and λex/em 535/617 nm for PI). About 12,000 events were acquired and positive FITC and/or PI cells were quantified by quadrant analysis and calculated using ACEA NovoExpress™ software (ACEA Biosciences Inc., San Diego, CA, USA). Each treatment was repeated three times and data represents means ± SEM of three replicates.

### Chemical composition of the plant extract

Liquid chromatography-mass spectrometry (LC-MS) was used to identify the major chemical compounds in the crude ethyl acetate extract of *P. juliflora *leaves. Chromatographic analysis was carried out by reverse phase elution (Waters Symmetry LC18 column 250 × 4.6 mm, 5 μm) on Agilent 6500 Series Accurate-Mass Quadrupole Time-of-Flight (Q-TOF; Agilent Santa Clara, CA, USA) LC/MS system with Agilent 1200 Series Diode Array Detector (module G1315B; detection type: 1024-element photodiode array; light source: deuterium and tungsten lamps; wavelength range 190-950 nm). The mobile phase consisted of (A) formic acid (0.1 %, v/v); (B) acetonitrile + 0.1 % formic acid; gradient (in solvent B): (i) 20 %, from 0 to 20 min, (ii) 95 %, from 20 to 27 min, and (iii) 35 %, at 27-30 min of total run time; flow rate: 0.2 ml/min; injection volume 3 L; ESI parameters: both negative and positive ion mode; mass range 100-1200 *m/z*; spray voltage 4 kV; gas temperature 325 °C; gas flow 10 L/min; Nebulizer 40 psi and the mass was analyzed by using Agilent technologies Mass-Hunter software.

### Statistical analysis

The IC_50_ for all cell lines was determined by ED_50_ plus V1.0 software. All data were expressed as mean ± standard error (SEM) of three replicates (*n *= 3). Statistical data were analyzed by *one-way* ANOVA and *Newman-Keuls* was used as *post-hoc* test. Graph-Pad Prism (V5, Co., San Diego, USA) was used for the statistical analysis. The differences between groups were considered significant at *P<0.05.

## Results

### Cytotoxic and anti-proliferative effects of P. juliflora extracts against cancer cell lines

**Figure 2:**
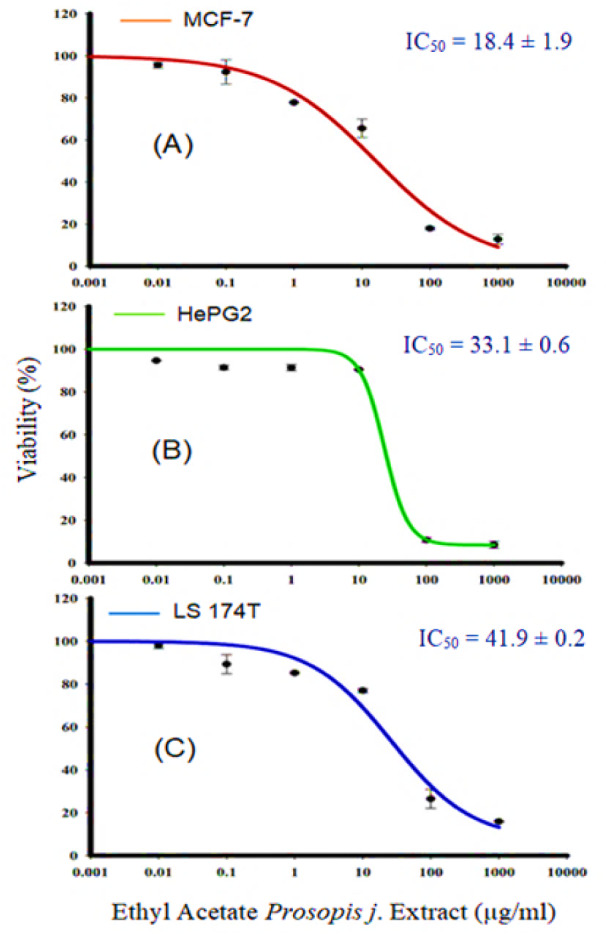
Effect of *P. juliflora* ethyl extracts on the viability of MCF-7 (A), HePG2 (B) and LS-174T (C) for breast, liver and colon cancer cell lines, respectively. Cells were exposed to extracts with the different concentrations for 72 h. Cell viability was determined after SRB stain. Data were expressed as mean ± SEM for three replicates (*n* = 3).

The goal of this experiment was to investigate the anti-proliferative and cytotoxic actions of *P. juliflora* leave extract against cancer cell lines. This allowed to identify the potential anti-cancer effect of *P. juliflora.* For this purpose, MCF-7, HepG2 as well as LS-174T cancer cells were first incubated for 72 hours with various concentrations (0.01 up to 1000 μg) of *P. juliflora *ethyl extracts, followed by cell viability assay and IC_50_ determination. Incubation with *P. juliflora *extracts showed concentration-dependent cytotoxicity against all tested cancer cell lines. The calculated IC_50_ was 18.17, 33.1 and 41.9 μg/ml for MCF-7, HePG2 and LS-174T, respectively (Table 1(Tab. 1), Figure 2(Fig. 2)). 

**Figure 3:**
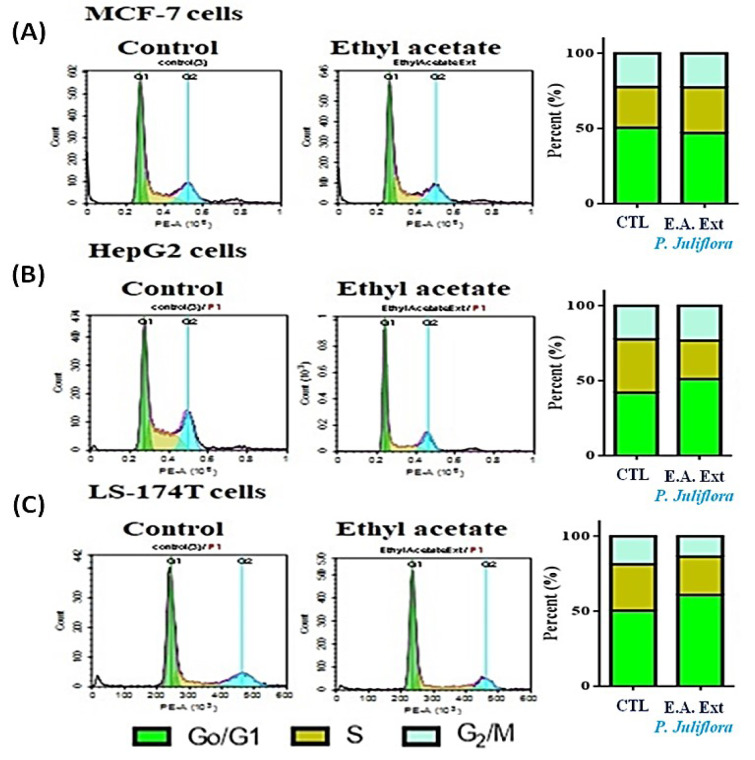
DNA cytometry analysis showing the effects of ethyl acetate extract of *Prosopis juliflora* on cell cycle distribution of MCF-7 (A), HePG2 (B) and LS-174T cells (C). Cells were exposed to ethyl acetate extract for 48 h. The different cell phases were plotted as percentage of total events. Sub-G cell population was plotted as percent of total events of cells. Data was presented as mean ± SEM for three replicates (*n*=3). The differences of events from each respective control were considered significant at *P<0.05.

In order to investigate the potential anti-proliferative effects of *P. juliflora *extracts against cancer cells, the aforementioned cell lines were incubated for 48 hours with the respective IC_50_ of *P. juliflora *extracts. Subsequently, the cell cycle phases were analyzed using DNA content flow cytometry. The plant leave extract arrested the cell cycle stages and exerted anti-proliferative effects in the tested cancer cell lines (Table 1(Tab. 1), Figure 3(Fig. 3)). The extract significantly decreased the G1-phase cell population in the MCF-7 with reciprocal increase of the S-phase cells (Table 1(Tab. 1), Figure 3A(Fig. 3)), but those of the later S-phase, were significantly decreased in both the HePG2 (Table 1(Tab. 1), Figure 3B(Fig. 3)) and the LS-174T cells (Table 1(Tab. 1), Figure 3C(Fig. 3)) in response to the plant extract with reciprocal increase of the G0/G1 phases compared to their respective controls (P<0.05). Interestingly, the plant extract also significantly decreased the G2/M-checkpoint cells in the LS-174T, indicating a strong anti-proliferative effect compared to control (Table 1(Tab. 1), Figure 3C(Fig. 3)). These results suggest that *P. juliflora *extracts induce cell specific anti-proliferative action.

**Figure 4:**
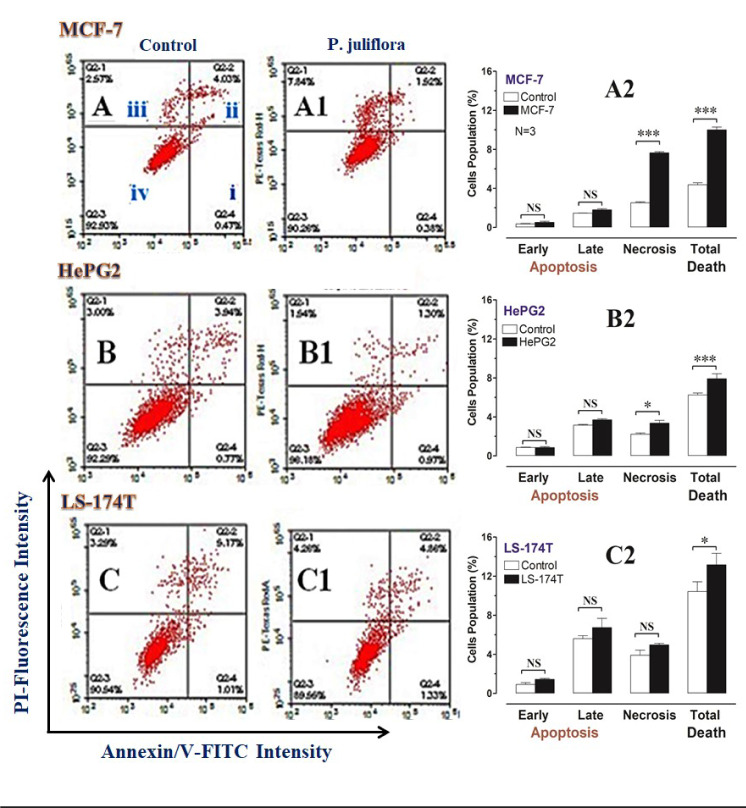
DNA cytometry analysis of Annexin V-FITC showing representative flow cytometry panels of apoptosis, necrosis and cell vitality of MCF-7, HePG2 and LS-174T in response to 48 h exposure to media only (control; CTL) (A, B, C, respectively), or ethyl acetate extract (E.A) of *Prosopis juliflora* (A1, B1, C1, respectively). The panel's quadrants represent; i) early apoptosis, ii) late apoptosis, iii) necrosis and iv) live cells. Cells population (%), including; early apoptosis, late apoptosis, necrosis and total cell death for the different treatments were shown in A2, B2 and C2, respectively. All data was presented as mean ± SEM for three replicates (*n*=3). Differences were considered significant at *P<0.05, ***P<0.001.

In order to identify the form of cell death induced by *P. juliflora *extracts, the tested cancer cell lines were incubated for 48 hours with the respective analysis using Annexin/V-FITC IC_50_ of the plant extracts. Subsequently, apoptotic and necrotic cells were differentiated by flow cytometry apoptosis detection assay. The ethyl acetate extracts of *P. juliflora* leaves did not induce significant apoptotic death in all tested cell lines compared to their respective controls (Table 2(Tab. 2), Figure 4(Fig. 4)). In contrast, the plant extract significantly increased the number of necrotic cells and the total cell death of both the MCF-7 and HePG2 cells population (Table 2(Tab. 2), Figure 4A, B(Fig. 4)) (P<0.05). Likewise, the plant extract non-significantly increased the necrotic cell population and total cell death of the LS-174T compared to their controls.

### Chemical composition of P. juliflora ethyl acetate extract

**Figure 5:**
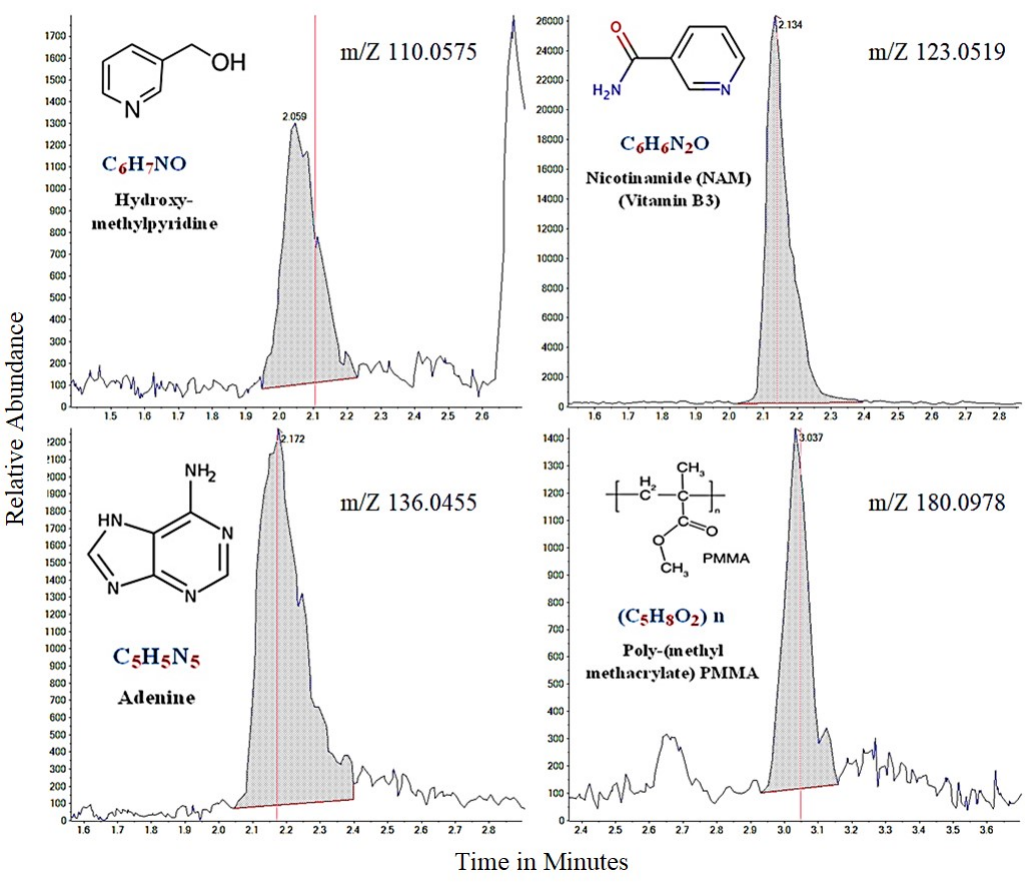
The major chemical compounds of *Prosopis juliflora* identified by the liquid-chromatography mass spectrometry (LC-MS)

The previous chapter shows that *P. juliflora* extracts have a potential anti-cancer effect against breast adenocarcinoma, hepatocellular carcinoma, and colorectal adenocarcinoma cell lines. In order to identify the anti-cancer constituents of *P. juliflora*, the chemical composition of the ethyl extracts was identified by using the liquid chromatography-mass spectrometry (LC-MS). The major chemical compounds identified in the ethyl extracts of *P. juliflora* leaves were hydroxymethyl-pyridine, nicotinamide (NAM; vitamin B3), adenine, and poly-(methyl methacrylate) (PMMA) (Table 2(Tab. 2), Figure 5(Fig. 5)). In conclusion, analysis of *P. juliflora* crude extract revealed enrichment of anti-cancer constituents.

## Discussion

In the present study we report a potential anti-cancer effect of *Prosopis juliflora* leave extract against three types of human cancer cell lines; MCF-7/breast, HePG2/liver and LS-174T/colorectal carcinomas. Breast cancer, hepatocellular carcinoma (HCC) and colorectal cancer (CRC) are frequent metastatic adenocarcinomas affecting human with higher mortalities. MCF-7 is widely used for *in vitro* breast cancer studies as a human breast adenocarcinoma with unique properties (Levenson and Jordan, 1997[22]). MCF-7 cells are fairly large adherent cells, with typical cell size of 20-25 microns, and their growth could be inhibited by the tumor necrosis factor alpha (TNF alpha) and the antiestrogens such as insulin-like growth factor-1 (IGF-1) (Fagan et al., 2017[8]; Venugopal et al., 2017[37]). There are eight HCC cell lines (Jung et al., 2012[18]); HepG2 is a human hepatoma of epithelial-like morphology commonly used in drug metabolism and hepatotoxicity studies (Donato et al., 2015[6]). HepG2 cells are characterized by higher rates of proliferation and secretion of a variety of plasma proteins, like albumin, transferrin, fibrinogen, alpha-2 macroglobulin, alpha-1 antitrypsin and plasminogen (Knowles et al., 1980[20]). Colorectal cancer is the 3^rd^ most common cancer in the world (Xu et al., 2015[38]). LS-174T was documented for production of high-molecular-weight mucins (Belley et al., 1996[2]). 

Breast cancer is the most leading cause of women death due to its serious malignancy (Siegel et al., 2015[32]) and still remains an incurable disease (Jamalzadeh et al., 2017[17]). Several studies proposed *P. juliflora* as a candidate anticancer agent (Jamalzadeh et al., 2017[17]). Based on the plant cytotoxicity and the present results of IC_50_ on the different cancer cells, it was clear that the plant leave extract showed higher cytotoxic specificity against the breast cancer cell line, MCF-7, rather than the other cancer cell lines; HePG2 and LS-174T, of liver and colorectal cancer, respectively. Our results were consistent to recent study reported that MCF-7 is highly sensitive to the killing effect of *P. juliflora* methanol extract that exerting low IC_50_; 19.4 μg/ml (Hassan et al., 2019[15]). The time, order and sequence of cell cycle events are monitored by the checkpoints occurred at the G1/S boundary, S-phase, and G2/M-phases (MacLachlan et al., 1995[23]). Those checkpoints are control systems which enable the cell to proliferate only in response to stimulatory signals as growth factors with serving the genetic information and hereditary among generations (Pucci et al., 2000[26]). They are activated by the DNA damage growth arrest at the mitotic spindle and so allow the cell to repair the damage and resume the cell cyclicity, but if the damage could not be repaired, the cell undergoes apoptosis (Pucci et al., 2000[26]). When DNA is damaged, cell cycle checkpoints indicate the proper execution of the cycle events in different pathways, as; a) blocking the cell entry into mitosis that is denoted by G2/M phase arrest (Taylor and Stark, 2001[34]), b) preventing DNA duplication as referred by S-phase arrest (Taylor and Stark, 2001[34]). However, the cell cycle arrest and apoptotic pathways depend on the cell type and the anticancer agent (Habtemariam, 2020[13]). Therefore, the ability of the different cancer cell lines; MCF-7, HePG2 and LS-174T, to release the mitochondrial or nucleic intrinsic factors in response to the tested plant extracts' compounds was variable in sensitivity. Ethyl extract of the *P. juliflora* leaves inhibited the cell growth and proliferation at G1, S- or G2/M phases in HePG2 and LS-174T, but not the MCF-7. 

Natural products extracted from plants could provide novel anticancer drugs (Shahali et al., 2018[30]). Likewise, our study presents the invasive plant; *Prosopis juliflora,* as a natural source of anticancer chemical compounds, i.e. hydroxy methylpyridine, nicotinamide (NAM), adenine and poly-(methyl methacrylate) (PMMA). All of those major compounds involved in *P. juliflora* were approved as anticancer agents in several studies. The cytostatic or cytotoxic properties of the hydroxy-methylpyridine were recently investigated by several studies (Radko et al., 2019[27]). Nicotinamide (NAM), the amide form of niacin (i.e. vitamin B3, endogenous metabolite) plays a major role in the metabolic pathway for production of nicotinamide adenine dinucleotide (NAD^+^) (Gasperi et al., 2019[10]). NAM further was found to arrest the cancer microenvironment by suppression of cancer associated fibroblasts (CAF) proliferation through increasing the cellular oxidative stress *in vitro* (Hassan et al., 2020[14]). Adenine is a purine derivative synthesized in human liver and also found in brewer's yeast and vegetables (Lai et al., 2019[21]) and detected in *P. juliflora*. It is pharmacologically used in several biological processes like cellular respiration, deoxyribonucleic acid (DNA) in protein synthesis and also as a component of the former nicotinamide (Lai et al., 2019[21]). Recently, adenine was strongly approved for inhibition of growth and proliferation of the colorectal cancerous cells through apoptotic and autophagic pathways of cell death (Lai et al., 2019[21]). Poly-methyl methacrylate (PMMA) is a biocompatible polyester with high resistance to chemical hydrolysis, high drug permeability and safety (Khan et al., 2019[19]). Furthermore, PMMA particles inhibited the cancer cell viability in highly selective and dose-dependent manner through nucleus condensation, augmentation and so disintegration (Siegel et al., 2015[32]). The HePG2 and LS-174T could be more sensitive to nicotinamide rather than those of MCF-7, and the later cells were more responsive to the antiproliferative agent poly-(methyl methacrylate) PMMA detected in the *P. juliflora* leaves extract.

## Conclusion

As per our knowledge, this is the first research report comparing the effects of ethyl acetate extract of *Prosopis juliflora* leaves on three types of human cancer cell lines; MCF-7/breast, HePG2/liver and LS-174T/colorectal carcinomas. The present study showed that ethyl acetate extract of *P. juliflora* leaves exerted higher cytotoxic effect on MCF-7 rather than HepG2 and LS-174T showing antiproliferative effects in different pathways throughout the cell stages. Furthermore, necrosis and increased total cell death were the most prominent effect detected with variant degrees of significance in the tested cells.

## Acknowledgements

The authors extend their appreciation to the Deanship of Scientific Research at King Khalid University for funding this work through Research Group Project (Number R.G.P.1/210/41).

## Conflict of interest

The authors declare that there is no conflict of interest.

## Figures and Tables

**Table 1:**
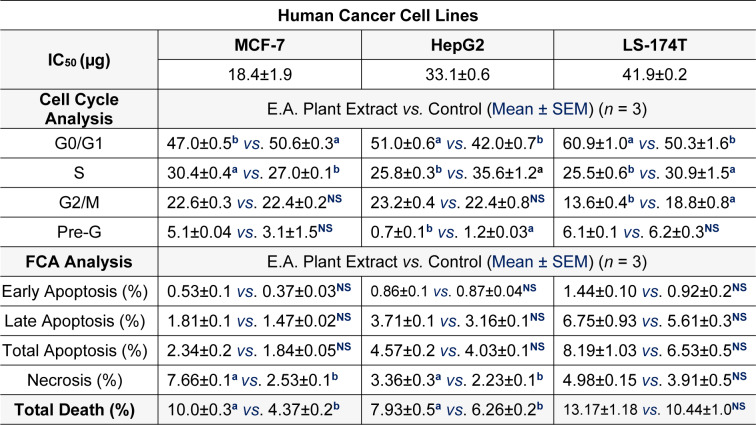
Half maximal inhibitory concentration (IC_50_) (µg) for ethyl acetate (E.A.) extract of *Prosopis juliflora* against the different human cancer; MCF-7/breast, HepG2/liver and LS-174T/colorectal cell lines. Effects of the ethyl-acetate extract of the plant against apoptotic and necrotic cell deaths were also shown. Letters; a, b, denote significant difference at P<0.05.

**Table 2:**
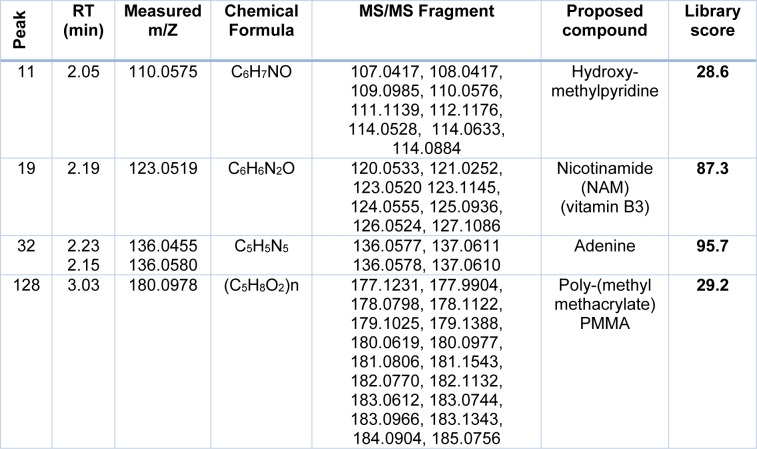
Chemical compounds identified in ethyl acetate extract of *Prosopis juliflora* by using the liquid chromatography mass spectrometry (LC-MS)

**Figure 1:**
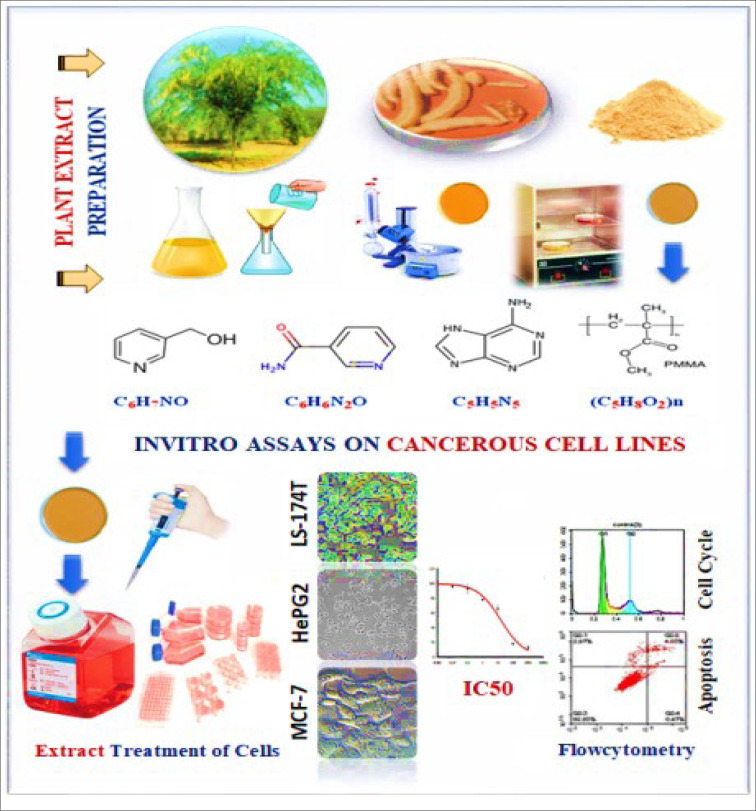
Graphical abstract
